# Effects of Transport Duration and Pre-Transport Fasting on Blood Biochemistry in Dorper × Mongolian Sheep

**DOI:** 10.3390/ani14101482

**Published:** 2024-05-16

**Authors:** Jin Xiao, Zhipeng Han, Xintong Li, Clive J. C. Phillips, Binlin Shi

**Affiliations:** 1 College of Animal Science, Inner Mongolia Agricultural University, Hohhot 010018, China; hzp4639@163.com (Z.H.); lixintongaa@163.com (X.L.); shibinlin@yeah.net (B.S.); 2 Curtin University Sustainability Policy (CUSP) Institute, Curtin University, Perth, WA 6845, Australia; clive.phillips@curtin.edu.au; 3 Institute of Veterinary Medicine and Animal Sciences, Estonian University of Life Sciences, Kreutzwaldi 1, 51014 Tartu, Estonia

**Keywords:** transport duration, pre-transport feeding, pre-transport fasting, blood indicators, summer, sheep

## Abstract

**Simple Summary:**

Transport is an unavoidable and important part of livestock husbandry. Live sheep transport is a period of high risk to their welfare, and potentially a huge hidden economic loss due to the effects on their health and/or the quality of their carcasses. Public concern about livestock transport indicates that it is important to improve the welfare of animals being transported. Prolonged transport and pre-transport fasting may particularly stress sheep, as well as potentially adversely affecting subsequent growth and/or carcass quality after slaughter. We investigated the effects of different transport times and pre-transport feeding during summer on the welfare of transported sheep of the Dorper × Mongolian breed, which are typical of the region and may be tolerant of the high temperatures in the Inner Mongolian summer. We selected 4-month-old male sheep to conduct two transport experiments, measuring the relevant blood biochemical indicators to evaluate the level of animal welfare. We found that three hours transport, compared with one hour, reduced the nutritional status of sheep, and that 12 h fasting before transport reduced the sheep’s nutritional status. Increased cortisol in sheep fed pre-transport may indicate that sheep are less stressed by the transport if they have been fasted. We therefore recommend that, during hot weather, sheep transport is confined to short periods. Regarding fasting, we recommend that sheep are not fasted for a long period beforehand, because of the decline in nutritional status.

**Abstract:**

Transport is a high-risk time for sheep, especially if the distances are long and sheep are fasted for a long time beforehand. Two experiments were conducted to compare transport durations of 1 hour (1 h) and 3 hours (3 h) and the effects of feeding before transport using Dorper × Mongolian sheep, which are typical of the region and may be tolerant of the high temperatures in the Inner Mongolian summer. Thirty 4-month-old male sheep were randomly divided into two treatment groups, with 15 sheep/treatment in each experiment, to evaluate the effects on blood biochemical indicators, stress hormone levels, rectal temperatures, and antioxidant status of lambs in summer. In Experiment 1, the levels of triglycerides and free fatty acids after 3 h transport were significantly lower than after 1 h transport (*p* < 0.05). The levels of thyroxine and malondialdehyde in blood were greater after 3 h transport than 1 h transport (*p* < 0.05). Creatine kinase levels after 3 h transport tended to be lower than after 1 h transport (*p* = 0.051). In Experiment 2, the levels of urea and superoxide dismutase in the group fasted pre-transport was significantly lower than those of the group fed pre-transport (*p* < 0.05). The serum cortisol level in the pre-transport fed group was higher compared to the group fed pre-transport (*p* = 0.04). Total antioxidant capacity in the pre-transport fasted group tended to be lower compared to that in the pre-transport fed group (*p* < 0.0001). We conclude that the reduction in nutritional status of sheep transported for longer and without feed pre-transport suggests that transporting sheep in hot conditions in northern China after fasting for a long period should be restricted. However, a decrease in the stress induced by transport following fasting is worthy of further study.

## 1. Introduction

Transport is an unavoidable and important part of livestock husbandry, during the introduction of new stock to the flock, off-site fattening, transport to the slaughterhouse, etc. With the development of the Chinese sheep industry, the age of lambs transported has been decreasing, but the distances travelled has been increasing [1]. Sheep lose weight during transport, particularly older sheep, and serum proteins, triglyceride, and cholesterol decrease while serum lipid oxidation increases [2]. Effects of transport on meat quality are dependent on age [2]. Transport stress is caused by a long transport duration, high environmental temperatures, poor road conditions and nutritional state, and a high stocking density [3,4,5]. Environmental temperature is one of the main causes of stress during transport [6]. Within the isothermal zone, body temperature is regulated through the neuroendocrine system to maintain a constant temperature [7]. When the temperature exceeds the thermoneutral zone, body temperature and/or physiology are affected, reducing their production performance and even endangering their lives [8]. Currently, 23 ± 2 °C is considered to be the thermoneutral temperature zone for sheep [9,10,11]. Transport, therefore, is a huge challenge for animal welfare [12]. During land transport in summer, a good ventilation system in vehicles reduces the Temperature Humidity Index (THI). When the driver stops, the THI is raised because of poor airflow [13]. The stop duration is directly proportional to the increase in THI during transport in summer [14]. The thermoneutral THI of sheep is estimated at 22.2 by Maria’s method [15]. Regarding the duration of transport, the most stressful period is at the start of the journey, but there is still greater stress on longer journeys than on short ones. Very long journeys may induce fatigue, depending on the ability of the animals to lie down, the facilities provided in the truck, and the quality of the road and driving [16].

It is usually not possible to provide water and feed during transport and, as the transport duration increases, the degree of hunger and thirst in animals magnifies, as do mental stress and metabolic disorders. Carnovale et al. [17] found that the stress levels during 1 h transport journeys were lower than those recorded during 2 h transport trips, and that fasting before transport increased transport stress in sheep during the Chinese winter. 

China has by far the largest number of sheep worldwide, and Inner Mongolia is the biggest producer of sheep meat of all the provinces in China [18]. Transport of sheep is mostly conducted in summer when maximum temperatures are typically 30–34 °C, above the normal upper critical temperature for sheep, potentially reducing feed intake and the metabolism of nutrients [19]. However, many farmers use Dorper or Dorper crosses, which are heat-stress-resistant [20].

Our hypotheses were that Inner Mongolian sheep would not be stressed by short journeys, but that longer journeys may prove stressful, and that fasting the sheep before transport will also reduce animal welfare. Our objective in this study was to evaluate the effects of transportation duration and pre-transport feeding on blood biochemical indicators, stress-related hormone levels, and antioxidant function of lambs in summer to provide a theoretical basis for the formulation of regional standards for the welfare of transported sheep in Inner Mongolia.

## 2. Materials and Methods

Sheep were selected so that the mean body weight in the two treatments of each experiment was similar. 

### 2.1. Experiment Location

The sheep were fed at 08:00 a.m. and 6:00 p.m. daily with the same ration: alfalfa hay (1.5 kg/head/d) first and then a pelleted feed (0.5 kg/head/d). Water was provided *ad libitum* both in the barn and in the outdoor area. They were transported in July 2021, and the start and the end of the transport was the Hailiutu experimental farm (location: 40°51′~41°8′ North latitude, 110°46′~112°10′ East longitude) of the Inner Mongolia Agricultural University, Hohhot, Inner Mongolia, China. All transportation took place around the middle of the day (10:00–13:00 h). The sheep were transported a total distance of 20 km on bitumen-surfaced roads (from 40°40′53″ N, 111°22′23″ E 1000 m height to 40°40′53″ N, 111°22′23″ E 1000 m height).

### 2.2. Experiment Design

Dorper × Mongolian sheep were selected for this study as they are typical of the region. Dorpers are known to be heat-stress-resistant [20] and have not been used in transport studies before, to the best of our knowledge. This is particularly relevant in the Inner Mongolian summer period when high temperatures can present a risk of heat stress. The number of sheep per treatment was determined by the size of trucks typically used in China for the transportation of sheep.

#### 2.2.1. Experiment 1; Transport Duration

This study compared two journeys, vehicle 1 was used for the 1 h journey and vehicle 2 was used for a 3 h journey on the same route. Thirty 4-month-old Dorper × Mongolian male sheep (wool length approximately 50 mm) with an average body weight of 30.80 ± 5.18 kg were selected and randomly divided into two groups. The two journeys used two identical vehicles: length 2.7 m, width 1.5 m, and axle height 0.32 m, 0.4 m solid walls with 1.1 m height cage, providing 0.27 m^2^/animal. The vehicles were driven on a return route on a straight, single lane carriageway (road numbers 038, 025, and 024) at a consistent speed of 60 km/h. The route was chosen so that there was minimal traffic, no traffic lights, and no stops. In order to minimize differences over time and between the journeys of the two vehicles, the two vehicles ran together at the same speed on the same route for the first 1 h. The condition of the remaining 2 h journey of the 3 h transport was similar to that of the first 1 h transport. At the last hour of the 3 h transport, the vehicle returned halfway to the starting point.

#### 2.2.2. Experiment 2; Pre-Transport Feeding vs. Fasting before Transport

Thirty 4-month-old Dorper × Mongolian male sheep (wool length approximately 50 mm), with an average body weight of 30.80 ± 5.18 kg were selected and randomly divided into two journeys. The first, fasted group had feed removed at 20:00 h of the night before they were transported and no feed was given until after transport completion.The second group that was fed before transport did not have feed removed at 20:00 h and were offered 0.5 kg pellets/head and 1.5 kg chopped alfalfa hay/sheep in a complete diet offered in a trough for one hour before transport. The nutrient levels of the feed DM, as declared by the manufacturer (CP feed), were as follows: digestible energy 11.12 MJ/kg, dry matter 90.72%, crude protein 13.62%, ether extract 1.96%, neutral detergent fiber 44.08%, acid detergent fiber 29.08%, ash 8.01%, Ca 0.83%, P 0.21%. The number of animals per treatment was determined by the size of the trucks typically used in China for the transportation of sheep. The sheep on both journeys were given free access to water before and after the journey. The same vehicles as those used for the 3 h journey in experiment 1 were used in this experiment, and the traffic speed and the route were the same.

### 2.3. Measurements

#### 2.3.1. Environmental Parameters

The ambient temperature, humidity, and wind speed at the local sheep farm were recorded at a local meteorological station throughout the day. The temperature on the day of transport was between 20 °C and 36 °C and the average relative humidity was 43%. The wind speed, temperature, and humidity were measured ten times with an anemometer (Testo 416 Digital Mini Vane Anemometer, 99 Washington Street, Melrose, MA 02176, USA) on vehicle 2 during transport in both experiments 1 and 2. 

#### 2.3.2. Rectal Temperature and Blood Indices

Immediately before and after the journeys, the rectal temperatures of sheep were measured, and two jugular blood samples were collected into heparinized tubes. Tubes were centrifuged for 20 min at 3000× *g* to collect the serum. The concentrations of catecholamines (CA), creatine kinase (CK), cortisol, heat shock protein (HSP70), insulin, triiodothyronine (T_3_), thyroxine (T_4_), and free fatty acids (FFA) in the serum were determined in accordance with the manufacturer’s instructions for the Elisa assay kits (Wuhan Genomics Technology Co., Ltd. Wuhan, China and Ruixin Biological Technology Co., Ltd. Quanzhou, China). Blood glucose, alkaline phosphatase (ALP), aspartate aminotransferase (AST), total protein (TP), albumin (ALB), total triglyceriodes (TG), total cholesterol (CHO), urea, alanine aminotransferase (ALT) and lactate dehydrogenase (LDH) were measured in accordance with the manufacturer’s instructions using the Lepu series of blood biochemical indicator reagents (Lepu, Lepu Medical Equipment Co., Ltd., Beijing, China) and a fully automated biochemical analyzer (HITACHI7020, Hitachi, Tokyo, Japan). The antioxidant indicators, total antioxidant capacity (T-AOC), glutathione peroxidase (GSH-Px), total superoxidase dismutase (T-SOD) enzyme activity, and malondialdehyde (MDA) content in serum were determined using the Nanjing Jiancheng Colorimetric Test Kit (Nanjing Jiancheng Institute of Bioengineering, Nanjing, China).

### 2.4. Statistical Analysis

Experiments were individually analyzed for statistical differences between treatments in experiments 1 and 2. Based on rectal temperatures and blood parameters measured before departure and after return, all the blood indices measured after return in Experiment 1 were compared between treatments using a general linear model of SAS 9.2 analysis software system for analysis of covariance (ANCOVA), including treatment (transport duration in experiment 1) as fixed effects and values at departure as the covariate. The experiment 2 data was analyzed using a GLM model of the SAS 9.2 analysis software system for a 2 × 2 two factor analysis of variance. The two factors in the model included pre-transport feeding (yes or no) and the sampling time (before and after transport).

Transport environmental indicators were reported as means and compared between the two treatments by student’s *t*-test in SAS 9.2 software in experiment 1. A *p*-value of <0.05 was considered to be statistically significant, 0.05 < *p*-value ≤ 0.10 were considered tendencies. Results are presented as means.

## 3. Results

### 3.1. Experiment 1; Transport Duration

#### 3.1.1. Environmental Parameters

**Figure 1:**
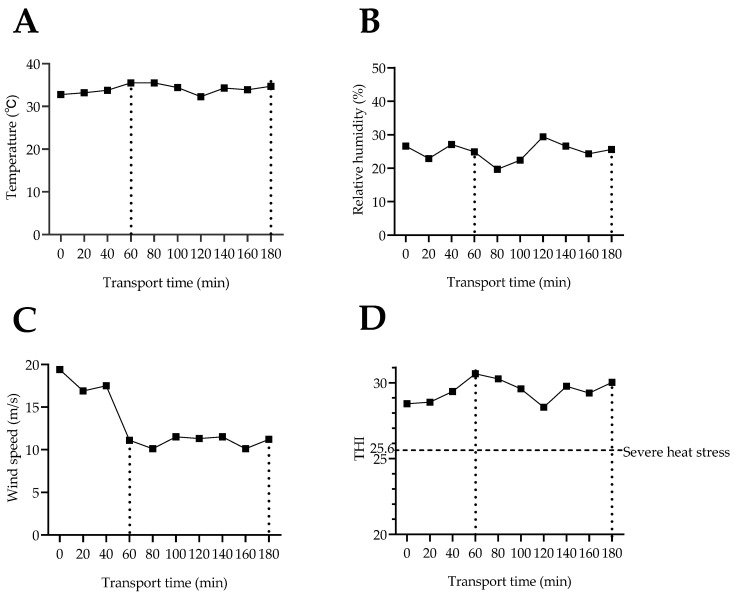
Transportation environmental indicators in experiment 1. (**A**) Transportation temperature; (**B**) relative humidity during transportation; (**C**) transportation wind speed; (**D**) Transportation THI. THI: Temperature–humidity index was calculated from the formula THI = db °C − [(0.31–0.31 RH) (db °C − 14.4)] [15], where db °C represents dry bulb temperature (°C) and RH represents relative humidity (%).

As shown in Table 1 and Figure 1, during the two journeys the environmental temperature was above 30 °C. The average temperature, humidity, and THI for the 1 h and 3 h transport groups were statistically the same (*p* > 0.100). The THI during the 1 h transport and 3 h transport was higher than 25.6, the threshold for entering a heat-stressed state [15]. 

#### 3.1.2. Rectal Temperature

Rectal temperature after transport was not significantly different between the 1 h and 3 h transport groups (*p* = 0.329) (Table 2).

#### 3.1.3. Serum Biochemical Indicators

As shown in Table 3, the levels of TG (*p* < 0.0001) and FFA (*p* < 0.05) after the 3 h transport were significantly lower than those recorded after the 1 h transport. T_4_ levels in the blood were significantly higher after the 3 h transport than after the 1 h transport (*p* < 0.0001). CK levels tended to be lower after the 3 h transport than after the 1 h transport (*p* = 0.051). The rest of the blood biochemical parameters did not reveal any statistical differences between the 1 h and 3 h transport groups. Serum antioxidant levels demonstrated increased levels of MDA in the 3 h treatment and a tendency for GSH-Px to be lower in this treatment (Table 4). 

### 3.2. Experiment 2; Pre-Transport Feeding vs. Fasting before Transport

#### 3.2.1. Environmental Parameters

**Figure 2:**
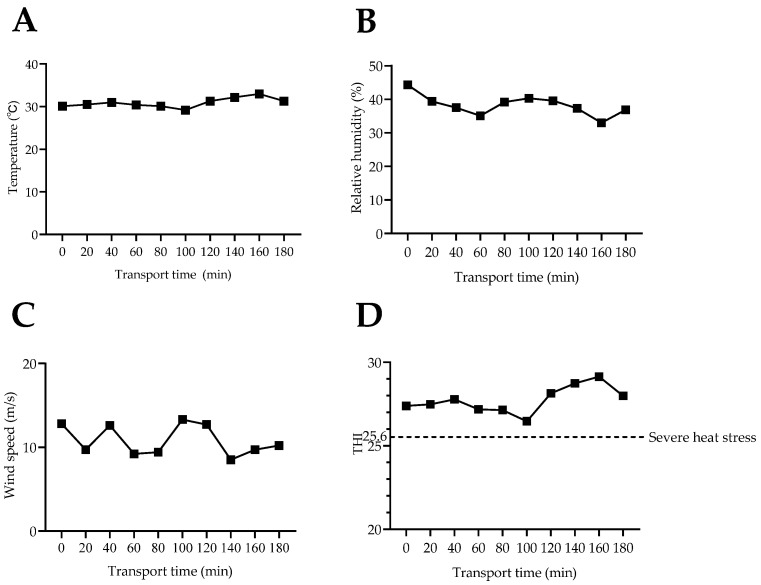
Transportation environmental indicators in experiment 2. (**A**) Transportation temperature; (**B**) relative humidity during transportation; (**C**) transportation wind speed; (**D**) transportation THI. THI: temperature–humidity index, the formula is: THI = db °C − [(0.31–0.31 RH) (db °C − 14.4)] [15], where db °C represents dry bulb temperature (°C) and RH represents relative humidity (%). The results are presented as means ± standard deviation.

As shown in Table 5 and Figure 2, during the journey, the environmental temperature was just in excess of 30 °C. The mean THI during transport was 27.74, which was higher than the 25.6 threshold for entering a heat-stressed state [15].

#### 3.2.2. Rectal Temperature

Rectal temperature after transport did not reveal statistical differences between the pre-transport feeding group and pre-transport fasting group (*p* = 0.298) (Table 6).

#### 3.2.3. Serum Biochemical Indicators

Urea was reduced at the end of the fasting period, but it recovered during the transport; in the non-fasted group, urea was reduced by the transport (Table 7). Insulin was reduced by fasting but recovered during the transport. TG was reduced and TP, glucose and CK increased by transport in both treatments. ALP and cortisol were increased in the fed group compared with the fasted group. 

#### 3.2.4. Serum Antioxidants

As shown in Table 8, T-SOD levels in the blood were significantly higher in the pre-transport feeding group than in the pre-transport fasting group, particularly at the end of the fast, before transport (*p* = 0.013). T-AOC was increased in the pre-transport feeding group both before and after the transport (*p* < 0.001). The levels of GSH-Px tended to be reduced after transport in both treatments, and MDA displayed no significant difference between the two treatment groups.

## 4. Discussion

### 4.1. Effects of Transport Duration on Blood Biochemical Indicators, Stress-Related Hormone Levels, and Antioxidant Function of Lambs in Summer

Transport causes stress and challenges to the welfare of sheep [21]. The poor welfare of animals during transportation can lead to the decline or stagnation of subsequent growth performance in animals, and the quality of meat products of animals experiencing transportation stress before slaughter will also decline. In order to improve animal welfare when sheep are transported, many international laws and standards refer to limiting the duration of sheep transportation, preferably to no more than 6 h. However, there have been few studies on the effects of short summer trips of less than four hours during hot weather on physiological parameters in sheep, and none assessing Dorper x Mongolian sheep, an important and potentially heat-stress-resistant breed in northern China. An increase in body temperature is commonly used to judge the stress state of production animals because it is one of the non-specific responses to stress. Rectal temperature represents the deep body temperature of an animal [22]. The documented THI > 25.6 suggests that the increased core body temperature was due to heat stress, but, in this study, neither the transport duration nor the pre-transport feeding status caused a change in the rectal temperature. Thus, it can be confirmed that the severity of transport stress was not enough to cause the changes in the sheep’s core temperature, probably due to the short transport duration both in experiment 1 and in experiment 2.

Triglycerides (TG) are the main energy storage substances in the body. Heat stress reduces TG levels in the blood, possibly due to the increased use of fatty acids for energy production in small, heat-stressed ruminants [23]. In this experiment, the levels of TG and FFA experienced a decrease by the end of the 3 h transport when compared to the 1 h transport, indicating that the longer transport duration raised energy consumption. Longer transport duration also upregulated T_4_ levels. This is probably because transport duration has a positive correlation with animal stress levels; for example, Kannan et al. [24] found that a 10 h transport resulted in greater stress in goats compared to a 2 h transport. Thyroid gland function is controlled by the HPT axis, and the thyroid gland mainly produces T_4_. Thyroid hormones are involved in meeting immediate energy demands and they act as metabolic integrators [25]. Recent experimental studies have focused on the crucial role of the HPT axis in energy homeostasis [26], also reporting that TRH hypophysiotropic neurons, considered to be metabolic sensors, are the central integrators of the HPT axis, decoding neuronal and hormonal signals related to energy status [27]. Therefore, the increase in T_4_ levels in the serum was consistent with the decrease in TG and FFA, an adaptive response after the sheep experienced being transported in hot conditions in this study.

Heat stress can have a series of effects on the behavior, physiology, endocrine, and molecular chaperones of ruminants [28]. The endocrine effects are mainly regulated by hormones secreted by the HPA axis and SAM axis. CK is the main acting hormone of the SAM axis, with increasing concentrations under heat stress [29]. CK levels in sheep tended to have decreased by the end of the 3 h transport compared to the 1 h transport in experiment 1. Creatine kinase is an important regulatory enzyme promoting the production of ATP by creatine phosphate (CP) in energy metabolism [30]. CK is positively correlated with the severity of stress [30]. Carnovale et al. [17] found that fasting before transportation caused significant transport stress in sheep during short distance transport. Therefore, the effect of transport duration on CK might be explained by an acute stress from the initial capture, blood collection, loading, and vehicle movement which was alleviated after the 3 h transport compared to the 1 h transport. For the 1 h transport, the sheep were captured and had blood collected twice within a one hour interval, whereas sheep in the 3 h transport had longer to recover from this stress.

Transport stress causes an imbalance in the antioxidant system in animals. MDA increased in the longer duration transport in our study. In another study, at 1 or 7 days after a 3 h transport, the MDA content in the serum was higher than that before transport in Simmental cattle [31]. Heat stress also increases the MDA content in the serum of dairy goats [32]. The above results indicate that both transportation and heat stress affect antioxidant function, a phenomenon which might be due to the stress increasing the degree of lipid peroxidation and MDA levels and a longer time required to return to normal lipid metabolism. In sheep, heat stress caused an increase in MDA content and a decrease in CAT enzyme activity in the serum, phenomena which were related to the Kelch-like ECH-associated protein-1/nuclear factor E2-related factor 2/antioxidant response element (Keap1/Nrf2/ARE) signaling pathway [33]. Heat stress suppresses the upstream hormone expression to reduce the gene expression of antioxidant enzymes through the Keap1/Nrf2/ARE pathway, thereby decreasing the activity of antioxidant enzymes in the serum [34]. In this study, the increase in MDA levels after the 3 h transport indicated that these sheep experienced more intracellular free radical aggregation and increased lipid peroxidation.

### 4.2. Effects of Pre-transport Feeding and Fasting before Transport on Blood Biochemical Indicators, Stress-Related Hormone Levels, and Antioxidant Function of Lambs in Summer

Fasting is normal before livestock transport, aiming to reduce the contents of the digestive tract of livestock during transportation to prevent animals from excreting excessive feces and urine into the transport vehicles to meet the hygiene requirements for roads and to improve carcass hygiene during slaughter [35,36]. However, studies have shown that fasting for a long duration before transportation can significantly reduce the carcass quality of sheep and, with the extension of fasting time, the degree of physiological stress also increases significantly [37]. In this study, cortisol levels experienced an increase in the fed group. This suggests that the sheep that travelled after fasting had reduced stress. It has been a contention of the livestock industry that the animals travel better empty, which is supported by this result. Transport itself causes stress: Alvarez et al. [38] found that the serum cortisol of cattle increases rapidly and then slowly decreases under long-term heat stress, findings which are consistent with the results of Abilay et al. [39]. The initial increase in concentration is due to the rapid activation of the HPA axis, while the gradual decrease after 2 h may be due to the initial adaptation of the organism to the stress. Broom et al. [40] conducted a study in which they collected blood samples every 30 min using a jugular vein catheter to monitor changes in the serum hormone levels in sheep during transport. They found that the changes in serum stress hormone levels, such as cortisol, mostly occurred in the first 3 h of transport; then, the changes in serum stress hormone levels were relatively small in the following 9 h. Carnovale et al. [17] found that fasting before transportation caused significant transport stress in sheep during short distance transport.

A decrease in serum urea levels with pre-transport fasting was evident in this study, possibly explained by the reduced energy status of the sheep during transport. TG was reduced by transport in both groups. TG is used to store energy following hydrolysis. Ruminants also depend on glucocorticoids to enhance the hydrolysis of TG in the fluid circulation for energy supply during heat stress [41]. Forhead et al. [42] found that fasting for 1–3 days before donkey transport increased cortisol and TG concentrations in serum. Smith et al. [43] established a mathematical model to predict the regulatory mechanism of the HPA axis by studying the concentration changes in arginine vasopressin (AVP) and CRH in the blood of sheep during road transport. The decrease in AVP and CRH during transport was due to a negative feedback effect of cortisol. Miranda-de et al. [44] indicate that significant transport stress increases the levels of cortisol in lambs.

Urea is an end product of protein metabolism. Transport has been shown to increase the serum urea in pigs which was utilized as an energy substrate consumed due to the stress [45]. When glycogen is depleted, proteins are used as a raw material for metabolic production [46]. One part of intracellular proteins enters mitochondria through the tricarboxylic acid cycle, synthesizing glucose for energy supply. The other part enters the urea cycle and, ultimately, is excreted from the body through sweating or urination [46]. Reduced urea in the fasted sheep was compensated by increased urea post-transport. Transport can increase urea levels (as shown in beef cattle by Ma et al. [47]), but, under heat stress conditions and without water to drink on the truck, this may be due to haemoconcentration.

Fasting affects the antioxidant capacity in animals [48], although in variable ways. The activity of SOD in muscles after 12 h fasting has been found to be significantly reduced in mice [49], agreeing with our observation of reduced T-SOD. However, in other studies, heat and transport have been found to increase serum SOD activity in cows [50] and serum SOD activity in beagle dogs has also been found to increase following a 2 h transport [51]. Water and feed supplementation to goats subjected to road transport effectively alleviates stress responses and any impact on antioxidant function [52]. The increase in SOD level caused by the pre-transport feeding indicates that nutrient intake was effective in enhancing antioxidant capacity. T-AOC was also increased by pre-transport feeding, which has been observed in transported fish [53].

These results can be utilized in educating veterinarians, technicians, and farmers in northern China about transport of sheep in hot conditions. The decrease in nutritional status and increased antioxidants following a 3 h transport, compared with a 1 h journey, suggest that, for sheep, journeys in hot weather should be restricted to short periods, or that consideration should be given to offering feed (and water) in the trucks which can be consumed either when the trucks are stationary or, on a smooth journey, during travel. Care would have to be taken that water provision does not result in wet, slippery floors. Further research is needed on the impact of fasting on stress levels, which were reduced in our study by fasting. 

### 4.3. Limitations of the Study

The study had several limitations which constrain the conclusions. The sample size was necessarily small to allow animals to be transported in vehicles typically used in China. However, it is an objective of ethical review that the minimum number of animals is used for the study and reusing animals between experiments would have assisted in this. Second, the three hours transport in experiment 1 continued after the first hour; therefore, the mean time at which the two groups were transported was not exactly the same, a factor which may have had an influence on hormones with circadian rhythms such as cortisol. It is also possible that different traffic conditions prevailed after the first hour, although unlikely given the nature of the road, or that the driving standards were different.

## 5. Conclusions

Body temperature, hormone levels, and blood biochemical parameters of sheep after transport for 1 h or 3 h in summer demonstrated that the longer transport duration reduced serum TG, FFA, T_4_, and CK and increased antioxidants MDA and GSH-Px, indicating a lower nutritional state. Fasting before transport reduced serum urea and TSOD, indicating that more protein was used for energy and that the sheep may have travelled better empty. Journey times should especially be limited during hot weather to short durations of no more than an hour or two. This will impose restrictions on transport of sheep during summer, but it is in the interest of the animals’ welfare and the quality of the products post-slaughter.

## Figures and Tables

**Table 1:** Transport environmental indicators in experiment 1.

Items	Temperature (°C)	Relative Humidity (%)	Wind Speed (m/s)	THI
Transport (1 h)	33.83	25.38	16.23	29.33
Transport (3 h)	34.04	24.95	13.06	29.47
SEM	0.35	0.82	1.14	0.25
*p* value	0.75	0.79	0.25	0.78

THI: temperature–humidity index was calculated from the formula: THI = db °C − [(0.31–0.31 RH) (db °C−14.4)] [15], where db °C represents dry bulb temperature (°C) and RH represents relative humidity (%).

**Table 2:** The influence of transport duration on sheep rectal temperature in summer. The results are presented as means (*n* = 15).

Items	Transport (1 h)	Transport (3 h)	SEM	*p*-Value
Rectal Temperature (°C)	39.75	39.89	0.09	0.329

**Table 3:** Effects of transport duration on serum biochemical parameters in sheep in summer.

Items	Transport (1 h)	Transport (3 h)	SEM	*p*-Value
ALP (U/L)	150.5	160.4	5.57	0.332
AST (mmol/L)	108.3	108.1	3.32	0.975
TP (g/L)	70.9	71.9	1.54	0.668
ALB (g/L)	39.3	39.3	0.91	0.981
TG (mmol/L)	0.49	0.33	0.01	<0.0001
CHO (mmol/L)	1.29	1.29	0.06	1.000
Urea (mmol/L)	11.06	10.70	0.31	0.590
Glucose (mmol/L)	7.52	7.07	0.42	0.469
LDH (U/L)	66.5	71.2	4.41	0.495
FFA (nmol/mL)	41.80	36.24	1.5	0.018
CA (ng/mL)	139.25	140.33	21.02	0.972
CK (mg/mL)	4.21	3.84	0.12	0.051
Cortisol (ng/mL)	19.51	17.84	0.71	0.117
HSP70 (ng/mL)	7.58	8.00	0.28	0.357
Insulin (mU/L)	6.22	5.70	0.39	0.424
TSH (pg/mL)	176.19	186.84	7.94	0.379
T_3_ (pg/mL)	275.04	297.71	15.23	0.406
T_4_ (ng/mL)	1.36	1.89	0.05	<0.0001

ALP: alkaline phosphatase, AST: aspartate aminotransferase, TP: total protein, ALB: albumin, TG: triglycerides, CHO: cholesterol, LDH: lactate dehydrogenase, FFA: free fatty acids, CA: catecholamine, CK: creatine kinase, HSP70: heat shock proteins 70, TSH: thyroid stimulating hormone, T_3_: triiodo–thyronine, T_4_: thyroxine. The results are presented as means (*n* = 15).

**Table 4:** Effects of transport duration on serum antioxidant indices of sheep in summer.

Items	Transport (1 h)	Transport (3 h)	SEM	*p*-Value
T-SOD (U/mL)	141.45	110.79	17.73	0.328
GSH-Px (U/mL)	104.40	72.13	12.86	0.095
MDA (nmol/mL)	0.16	0.28	0.03	0.006
T-AOC (mM)	0.94	0.89	0.02	0.110

T-SOD: total superoxidase dismutase, GSH-Px: glutathione peroxidase, MDA: malondialdehyde, T-AOC: total antioxidant capacity. The results are presented as means (*n* = 15).

**Table 5:** Transport environmental indicators in experiment 2.

Items	Temperature (°C)	Relative Humidity (%)	Wind Speed (m/s)	THI
Transport	30.91	38.26	10.81	27.74

THI: temperature–humidity index, the formula is: THI = db °C − [(0.31–0.31 RH) (db °C − 14.4)] [15], where db°C represents dry bulb temperature (°C) and RH represents relative humidity (%). The results are presented as means. (*n* = 15).

**Table 6:** The influence of pre-transport feeding and fasting before transport in summer of sheep on post-transport rectal temperature. The results are presented as means (*n* = 15).

Items	Pre-Transport Feeding	Pre-Transport Fasting	SEM	*p*-Value
Rectal Temperature (°C)	39.69	39.79	0.06	0.298

**Table 7:** Effects of Pre-transport feeding and fasting before transport on blood biochemical parameters in sheep in summer.

Items	Pre-Transport Fasting		Pre-Transport Feeding		SEM	*p*-Value		
	Before	After	Before	After		Pre-Transport Feeding	Sampling Time	Pre-Transport Feeding × Sampling Time
ALP (U/L)	161.1	143.9	190.6	180.3	13.78	0.026	0.338	0.808
AST (mmol/L)	125.7	131.2	117.6	120.3	9.05	0.301	0.653	0.878
TP (g/L)	68.2	73.4	71.1	73.9	1.74	0.334	0.027	0.494
ALB (g/L)	37.4	40.1	38.6	39.1	0.91	0.913	0.088	0.235
TG (mmol/L)	0.42	0.37	0.48	0.36	0.02	0.258	<0.0001	0.118
CHO (mmol/L)	1.37	1.38	1.21	1.20	0.06	0.007	0.960	0.893
Urea (mmol/L)	8.93	9.57	9.74	8.64	0.30	0.844	0.435	0.006
Glucose (mmol/L)	5.18	6.16	5.35	6.41	0.33	0.523	0.004	0.905
LDH (U/L)	61.4	63.4	63.6	65.9	4.92	0.636	0.669	0.975
FFA (nmol/mL)	36.4	38.1	39.2	36.6	1.46	0.656	0.741	0.149
CA (ng/mL)	142.3	115.9	128.9	120.0	13.63	0.737	0.205	0.525
CK (mg/mL)	3.91	4.14	3.70	3.98	0.12	0.129	0.038	0.815
Cortisol (ng/mL)	18.09	17.08	19.33	19.41	0.83	0.039	0.579	0.515
HSP70 (ng/mL)	7.57	6.91	7.83	7.43	0.44	0.384	0.237	0.779
Insulin (mU/L)	5.22	6.72	6.88	6.51	0.28	0.014	0.052	0.002
TSH (pg/mL)	191.5	179.1	198.6	192.7	10.84	0.345	0.403	0.765
T_3_ (pg/mL)	291.4	268.6	278.8	269.3	10.67	0.583	0.139	0.541
T_4_ (ng/mL)	1.60	1.63	1.70	1.55	0.06	0.848	0.310	0.122

ALP: alkaline phosphatase, AST: aspartate aminotransferase, TP: total protein, ALB: albumin, TG: triglycerides, CHO: cholesterol, LDH: lactate dehydrogenase, FFA: free fatty acids, CA: catecholamine, CK: creatine kinase, HSP70: heat shock proteins 70, TSH: thyroid stimulating hormone, T_3_: triiodo–thyronine, T_4_: thyroxine. The results are presented as means (*n* = 15).

**Table 8:** Effects of pre-transport feeding or fasting before transport on serum antioxidant indices of sheep in summer.

Items	Pre-Transport Fasting		Pre-Transport Feeding		SEM	*p*-Value		
	Before	After	Before	After		Pre-Transport Feeding	Sampling Time	Pre-Transport Feeding × Sampling Time
T-SOD (U/mL)	87.80	150.50	164.20	160.10	12.72	0.002	0.027	0.013
GSH-Px (U/mL)	179.80	128.90	146.90	133.20	16.27	0.385	0.055	0.260
MDA (nmol/mL)	0.22	0.26	0.28	0.20	0.05	0.501	0.583	0.819
T-AOC (mM)	0.80	0.77	0.87	0.88	0.02	<0.0001	0.492	0.324

T-SOD: total superoxidase dismutase, GSH-Px: glutathione peroxidase, MDA: malondialdehyde, T-AOC: total antioxidant capacity, Before: Before the transport, After: After the transport. The results are presented as means (*n* = 15).

## Data Availability

Data are available from the first author on request.
